# Type I collagen extracellular matrix facilitates nerve regeneration via the construction of a favourable microenvironment

**DOI:** 10.1093/burnst/tkae049

**Published:** 2024-12-10

**Authors:** Panjian Lu, Zhiying Chen, Mingjun Wu, Shuyue Feng, Sailing Chen, Xiyang Cheng, Yahong Zhao, Xingyu Liu, Leilei Gong, Lijing Bian, Sheng Yi, Hongkui Wang

**Affiliations:** Key Laboratory of Neuroregeneration of Jiangsu and Ministry of Education, Co-innovation Center of Neuroregeneration, NMPA Key Laboratory for Research and Evaluation of Tissue Engineering Technology Products, Medical School of Nantong University, Nantong University, 19 Qixiu Road, Nantong, Jiangsu 226001, China; Key Laboratory of Neuroregeneration of Jiangsu and Ministry of Education, Co-innovation Center of Neuroregeneration, NMPA Key Laboratory for Research and Evaluation of Tissue Engineering Technology Products, Medical School of Nantong University, Nantong University, 19 Qixiu Road, Nantong, Jiangsu 226001, China; Key Laboratory of Neuroregeneration of Jiangsu and Ministry of Education, Co-innovation Center of Neuroregeneration, NMPA Key Laboratory for Research and Evaluation of Tissue Engineering Technology Products, Medical School of Nantong University, Nantong University, 19 Qixiu Road, Nantong, Jiangsu 226001, China; Key Laboratory of Neuroregeneration of Jiangsu and Ministry of Education, Co-innovation Center of Neuroregeneration, NMPA Key Laboratory for Research and Evaluation of Tissue Engineering Technology Products, Medical School of Nantong University, Nantong University, 19 Qixiu Road, Nantong, Jiangsu 226001, China; Key Laboratory of Neuroregeneration of Jiangsu and Ministry of Education, Co-innovation Center of Neuroregeneration, NMPA Key Laboratory for Research and Evaluation of Tissue Engineering Technology Products, Medical School of Nantong University, Nantong University, 19 Qixiu Road, Nantong, Jiangsu 226001, China; Key Laboratory of Neuroregeneration of Jiangsu and Ministry of Education, Co-innovation Center of Neuroregeneration, NMPA Key Laboratory for Research and Evaluation of Tissue Engineering Technology Products, Medical School of Nantong University, Nantong University, 19 Qixiu Road, Nantong, Jiangsu 226001, China; Key Laboratory of Neuroregeneration of Jiangsu and Ministry of Education, Co-innovation Center of Neuroregeneration, NMPA Key Laboratory for Research and Evaluation of Tissue Engineering Technology Products, Medical School of Nantong University, Nantong University, 19 Qixiu Road, Nantong, Jiangsu 226001, China; Key Laboratory of Neuroregeneration of Jiangsu and Ministry of Education, Co-innovation Center of Neuroregeneration, NMPA Key Laboratory for Research and Evaluation of Tissue Engineering Technology Products, Medical School of Nantong University, Nantong University, 19 Qixiu Road, Nantong, Jiangsu 226001, China; Key Laboratory of Neuroregeneration of Jiangsu and Ministry of Education, Co-innovation Center of Neuroregeneration, NMPA Key Laboratory for Research and Evaluation of Tissue Engineering Technology Products, Medical School of Nantong University, Nantong University, 19 Qixiu Road, Nantong, Jiangsu 226001, China; Imperial College of Science, Technology and Medicine, London SW7 2AZ, United Kingdom; Key Laboratory of Neuroregeneration of Jiangsu and Ministry of Education, Co-innovation Center of Neuroregeneration, NMPA Key Laboratory for Research and Evaluation of Tissue Engineering Technology Products, Medical School of Nantong University, Nantong University, 19 Qixiu Road, Nantong, Jiangsu 226001, China; Key Laboratory of Neuroregeneration of Jiangsu and Ministry of Education, Co-innovation Center of Neuroregeneration, NMPA Key Laboratory for Research and Evaluation of Tissue Engineering Technology Products, Medical School of Nantong University, Nantong University, 19 Qixiu Road, Nantong, Jiangsu 226001, China

**Keywords:** Extracellular matrix, Collagen I, Collagen IV, Regenerative microenvironment, Peripheral nerve regeneration, Scaffold, Chitosan nerve conduit

## Abstract

**Background:**

The extracellular matrix (ECM) provides essential physical support and biochemical cues for diverse biological activities, including tissue remodelling and regeneration, and thus is commonly applied in the construction of artificial peripheral nerve grafts. Nevertheless, the specific functions of essential peripheral nerve ECM components have not been fully determined. Our research aimed to differentially represent the neural activities of main components of ECM on peripheral nerve regeneration.

**Methods:**

Schwann cells from sciatic nerves and neurons from dorsal root ganglia were isolated and cultured *in vitro*. The cells were seeded onto noncoated dishes, Matrigel-coated dishes, and dishes coated with the four major ECM components fibronectin, laminin, collagen I, and collagen IV. The effects of these ECM components on Schwann cell proliferation were determined via methylthiazolyldiphenyl-tetrazolium bromide (MTT), Cell Counting Kit-8, and 5-ethynyl-2'-deoxyuridine (EdU) assays, whereas their effects on cell migration were determined via wound healing and live-cell imaging. Neurite growth in neurons cultured on different ECM components was observed. Furthermore, the two types of collagen were incorporated into chitosan artificial nerves and used to repair sciatic nerve defects in rats. Immunofluorescence analysis and a behavioural assessment, including gait, electrophysiology, and target muscle analysis, were conducted.

**Results:**

ECM components, especially collagen I, stimulated the DNA synthesis and movement of Schwann cells. Direct measurement of the neurite lengths of neurons cultured on ECM components further revealed the beneficial effects of ECM components on neurite outgrowth. Injection of collagen I into chitosan and poly(lactic-co-glycolic acid) artificial nerves demonstrated that collagen I facilitated axon regeneration and functional recovery after nerve defect repair by stimulating the migration of Schwann cells and the formation of new blood vessels. In contrast, collagen IV recruited excess fibroblasts and inflammatory macrophages and thus had disadvantageous effects on nerve regeneration.

**Conclusions:**

These findings reveal the modulatory effects of specific ECM components on cell populations of peripheral nerves, reveal the contributing roles of collagen I in microenvironment construction and axon regeneration, and highlight the use of collagen I for the healing of injured peripheral nerves.

## Graphical Abstract

**Graphical Abstract:**
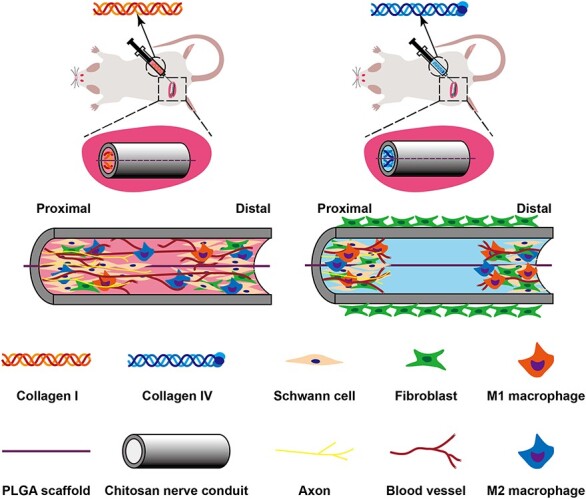

Highlights

- ECM components support Schwann cell growth, stimulate Schwann cell movement, and promote neurite growth *in vitro*.
- Type I collagen promotes axon regeneration and functional recovery by enhancing Schwann cell migration and vascularisation *in vivo*.
- Type IV collagen-induced fibroblast accumulation and excessive inflammation can be detrimental to peripheral nerve regeneration *in vivo*.

## Background

The extracellular matrix (ECM) is a noncellular, 3D meshwork that provides essential physical and mechanical support for tissue integrity and elasticity [1,2]. The ECM is mainly composed of fibrous proteins, adhesive glycoproteins, and glycosaminoglycans [3]. Although the basic components of the ECM are consistent, each organ has its own unique composition as the ECM is deposited and assembled by surrounding cells [1]. Unique components of the ECM offer critical mechanical inputs and biochemical cues for the regulation of various physiological and pathological processes [4–6]. Moreover, recent studies have demonstrated that the ECM induces cellular reprogramming, linking ECM remodelling to tissue regeneration [7,8].

Peripheral nerve injury is a universal clinical problem with a high disability rate. Satisfactory functional regeneration of peripheral nerve defects with long nerve gaps requires the implantation of autologous nerve grafts or neural scaffolds [9]. Given the fundamental roles of native ECM in neural regeneration, cell-derived ECM has been used to bridge peripheral nerve gaps [10–13]. The peripheral nerve ECM is primarily produced by Schwann cells and is chiefly composed of the glycoproteins fibronectin, laminin, tenascin, and collagen, as well as proteoglycans perlecan, agrin, and chondroitin sulfate proteoglycans [14]. Many peripheral nerve ECM components, such as fibronectin, laminin, and collagen, have been directly used to generate or modify engineered nerve grafts [15–18]. These ECM component-constructed nerve grafts modulate the behaviour of Schwann cells and neurons and facilitate peripheral nerve repair [19]. Despite the recognition of the beneficial effects of ECM components on peripheral nerve regeneration, the specific biological function of each peripheral nerve ECM component has not been fully explored and which component plays a superior role remains unknown.

Here, we coated culture dishes with fibronectin, laminin, collagen I, or collagen IV, four major components of the peripheral nerve ECM; cultured Schwann cells and neurons on coated dishes; and examined the effects of these ECM components on these major cell populations of peripheral nerves. Matrigel, a basement membrane matrix extracted from Engelbreth–Holm–Swarm mouse sarcoma containing ~60% laminin, 30% collagen IV, 8% nidogen-1/entactin, heparan sulfate proteoglycans, and a number of growth factors, such as transforming growth factor-beta 1, epidermal growth factor, and insulin-like growth factor was used as a positive control because of its beneficial effects during cell culture [20]. Collagen I and collagen IV, two ECM components with beneficial roles on cultured cells, were further injected into chitosan and poly(lactic-co-glycolic acid) (PLGA) scaffolds to determine their *in vivo* effects on peripheral nerve regeneration.

## Methods

### Cell culture

The sciatic nerves and dorsal root ganglias of neonatal Sprague–Dawley (SD) rats were isolated, surgically excised, and subjected to enzyme treatment to collect cell populations. The sciatic nerve was isolated from neonatal SD rats (1 day old), and 1 ml of collagenase (Gibco, Grand Island, NY, USA) was added at a concentration of 3 mg/ml. The tissue was minced using microscissors, and then the tissue suspension was placed into a cell culture incubator for digestion at 37°C for 30 min. Following centrifugation at 1200 rpm for 5 min, the supernatant was discarded, and 1 ml of trypsin (Gibco) was added at a concentration of 0.125%. The digestion process continued in the cell culture incubator for an additional 10 min, after which dulbecco's modified eagle medium (DMEM) (10–013-CVR, Corning, NY, USA) containing 10% fetal bovine serum (FBS) (Gibco) was added to terminate the enzymatic digestion. The cells collected from sciatic nerves were treated with anti-Thy1.1 (Sigma, St. Louis, MO, USA) and rabbit complement (Invitrogen, Carlsbad, CA, USA) to remove fibroblasts and collect Schwann cells. Schwann cells were maintained in DMEM supplemented with 10% FBS, 1% penicillin and streptomycin (Beyotime, Shanghai, China), 2 μM forskolin (Sigma), and 10 ng/ml β-heregulin (R&D Systems Inc., Minneapolis, MN, USA). The dorsal root ganglia were isolated from neonatal SD rats (1 day old) and 1 ml of collagenase was added at a concentration of 3 mg/ml. The samples were incubated in a cell culture incubator at 37°C for 30 min to digest the tissue. Following digestion, the collagenase was discarded and then 1 ml of trypsin at a concentration of 0.125% was added. The mixture was incubated in a cell culture incubator at 37°C for 20 min. To terminate the digestion, DMEM containing 10% FBS was added. The cells collected from the dorsal root ganglia were suspended in 15% bovine serum albumin (BSA) and centrifuged to collect pellets containing neurons. The neurons were maintained in neurobasal medium (Gibco) supplemented with 2% B27 supplement (Gibco), 2 mM L-glutamine (Thermo Fisher Scientific, Waltham, MA, USA), and 1% penicillin and streptomycin (Invitrogen).

### MTT assay

Flat bottom 96-well plates were coated with 10 or 100 μg/ml Matrigel (Corning), fibronectin (Millipore, Temecula, CA, USA), laminin (Corning), collagen I (Corning), or collagen IV (Corning) dissolved in DMEM for 2 h and then washed with PBS three times. A total of 1 × 10^4^ Schwann cells were suspended in 200 μl of culture medium and seeded onto 96-well plates. The cell growth conditions were measured via the methylthiazolyldiphenyl-tetrazolium bromide (MTT) assay. Briefly, after exposure to 20 μl of 5 mg/ml MTT (Sigma) for 4 h, the cell culture was terminated and the supernatant was discarded. Next, 150 μl of dimethyl sulfoxide (DMSO) was added to dissolve the MTT formazan crystals. The absorbance at 490 nm was recorded via a microplate reader.

### Cell viability assay

A total of 2 × 10^4^ Schwann cells were suspended in 100 μl of culture medium and seeded onto noncoated or precoated 96-well plates for 24 h. A total of 10 μl of Cell Counting Kit-8 (CCK-8; Beyotime) solution was added to the cell culture medium, and the mixture was incubated for 2 h according to the manufacturer’s instructions. The absorbance at 450 nm was recorded via a microplate reader.

### EdU incorporation assay

A total of 2 × 10^4^ Schwann cells were suspended in 100 μl of culture medium, seeded onto noncoated or precoated 96-well plates, and subjected to an 5-ethynyl-2'-deoxyuridine (EdU) incorporation assay via a Cell-Light EdU DNA Cell Proliferation Kit (RibiBio, Guangzhou, Guangdong, China). Briefly, 50 μM EdU was added to the cell culture medium after the cells adhered. The cells were cultured for an additional 12 h, fixed with 4% formaldehyde and stained with EdU and Hoechst 33342. Images were obtained with a fluorescence microscope (Leica Microsystems, Bensheim, Germany).

### Wound healing assay

A total of 2 × 10^4^ Schwann cells were suspended in 100 μl of culture medium and seeded onto noncoated or precoated 6-well plates. Wound healing culture inserts (ibidi, Martinsried, Germany) were placed on culture dishes and removed after the cells reached confluence. The cells were allowed to grow for ~10 h after insert removal. Images were obtained with an inverted microscope. The cleaned area was measured using Image-Pro Plus (Media Cybernetics, Silver Spring, MD, USA).

### Live-cell imaging

A total of 2 × 10^4^ Schwann cells were suspended in 200 μl of culture medium and seeded onto noncoated or precoated Nunc™ Lab-Tek™ chambers (Thermo Fisher Scientific). After the Schwann cells were cultured in a humidified 5% CO_2_ incubator at 37°C for 30 min, the chambers were placed on an Olympus IX81 microscope (Olympus, Tokyo, Japan). Time-lapse images were taken every 5 min for 11 h. Movement distance and cellular velocity were measured using Xcellence Sequence (Olympus) and ImageJ (National Institutes of Health, Bethesda, MD, USA).

### Animal surgery and tissue collection

Adult male SD rats weighing 180–220 g were deeply anaesthetised via an intraperitoneal injection of pentobarbital sodium and subjected to sciatic nerve resection as previously described [21]. Briefly, rat sciatic nerves at the left mid-thigh were exposed, and an 8-mm nerve segment was removed to generate a 10-mm nerve gap after spontaneous retraction of nerve stumps. After bridging the nerve gap with the chitosan and PLGA scaffolds [21], collagen I (1000 μg/mL), collagen IV (1000 μg/mL), or saline was injected into the conduits and the muscle layers and skin were closed with sutures. The rats were perfused with normal saline and 4% paraformaldehyde, and sciatic nerve grafts were collected 14 days after nerve bridging. For the *in vivo* examination of cell proliferation, 10 mM EdU (C10340, Invitrogen) was dissolved in 500 μl of saline and intraperitoneally injected into the rats 24 h prior to nerve graft collection.

### Immunostaining

Neurons cultured on noncoated or precoated 96-well plates for 36 h were fixed with 4% paraformaldehyde and incubated with primary anti-beta III tubulin (1: 1000, Abcam) and secondary antibody goat anti-rabbit immunoglobin G (IgG)-cyanine 3 (Cy3 ) (1:400, Proteintech). Images were obtained with a Zeiss Axio Imager M2 (Carl Zeiss Microscopy GmbH). Neurite length was determined via ImageJ. Sciatic nerve grafts were fixed, cut into 12-μm-thick slices, and incubated with primary antibodies: mouse anti-NF200 (1 : 200; Sigma), rabbit anti-S100 (1 : 200; Abcam, Cambridge, Massachusetts, USA), goat anti-CD34 (1 : 50; R&D), mouse anti-P4HB (1 : 100; Abcam), mouse anti-CD68 (1 : 100; Abcam), rabbit anti-CD206 (1 : 100; Abcam), rabbit anti-Iba1 (1 : 250; Wako), and mouse anti-inducible nitric oxide synthase (iNOS) (1 : 100; Santa Cruz Biotechnology) and secondary antibodies goat anti-mouse IgG H&L (Alexa Fluor® 488) (1 : 500; Abcam), sheep anti-rabbit IgG-Cy3 (1 : 1000; Sigma), and donkey anti-goat IgG H&L (Alexa Fluor® 647) (1 : 200; Abcam). The nuclei were immunostained with Hoechst 33342 (1 : 5000; Life Technologies, Carlsbad, CA, USA). Images were captured with a Zeiss Axio Imager M2 microscope. Quantitative analysis was performed via ImageJ and AngioTool software [22].

### Gait analysis

Four weeks after the surgery, the integrated functional recovery of the rats was assessed via Catwalk XT 9.0 (Noldus, Wageningen, The Netherlands) gait analysis system. The animals were allowed to pass through a glass walkway, underneath which a video camera captured each run. Paws were captured and 3D footprint intensities were measured. Max contact area was the maximum area of a paw that came into contact with the glass plate. Max contact max intensity was the maximum intensity at max contact of a paw. The sciatic functional index (SFI) was calculated to reflect functional recovery. SFI = −38.3[(PL_E_ − PL_N_)/PL_N_] + 109.5[(TS_E_ − TS_N_)/TS_N_] + 13.3[(ITS_E_ − ITS_N_)/IT_N_] − 8.8 (where PL is manual print length, TS is toe spread, ITS is intermediate toe spread and the subscripts E and N indicate the experimental and normal contralateral hind paws, respectively). An SFI value of −100 indicated abnormal sciatic nerve function, whereas an SFI value of 0 indicated normal sciatic nerve function.

### Electrophysiology analysis

Electrophysiological examinations were performed on the rats 4 weeks after surgery. Under deep anaesthesia, we re-exposed the sciatic nerve, including the bridging segment, on the surgical side of each rat. An electrical stimulus of 5 mA intensity was applied to the proximal sciatic nerve trunk of the graft via a Keypoint 2 portable electromyograph (Dantec, Denmark), and the compound muscle action potentials (CMAPs) of the gastrocnemius muscle were recorded.

### Gastrocnemius wet muscle weight

Under deep anaesthesia, the bilateral gastrocnemius muscles were extracted from the rats four weeks after surgery and subsequently weighed. Muscle mass is a reliable indicator of nerve reinnervation; the muscle weight was collected and the wet weight ratio was calculated.

### Bioinformatic analysis

The sequencing data of rat sciatic nerves at 0, 1, 4, 7, and 14 days after nerve crush injury were downloaded from the national center for biotechnology information (NCBI) database under accession number PRJNA394957 (SRP113121) [23,24]. The reads per kilobase transcriptome per million mapped reads (RPKM) method was applied to determine the mRNA abundances of the ECM-related genes. Gene expression patterns were displayed in a heatmap using Multiple Experiment Viewer (MeV) software.

### Statistics

The data were tested via the Kolmogorov–Smirnov (KS) normality test and are presented as the means ± SDs. Graphs and statistical analyses were conducted via GraphPad Prism software (GraphPad Software Inc., La Jolla, CA, USA). One-way analysis of variance (ANOVA) followed by Dunnett’s multiple comparisons test was used for multiple group comparisons. A *P* value < 0.05 was considered statistically significant.

## Results

### ECM components support Schwann cell growth

**Figure 1:**
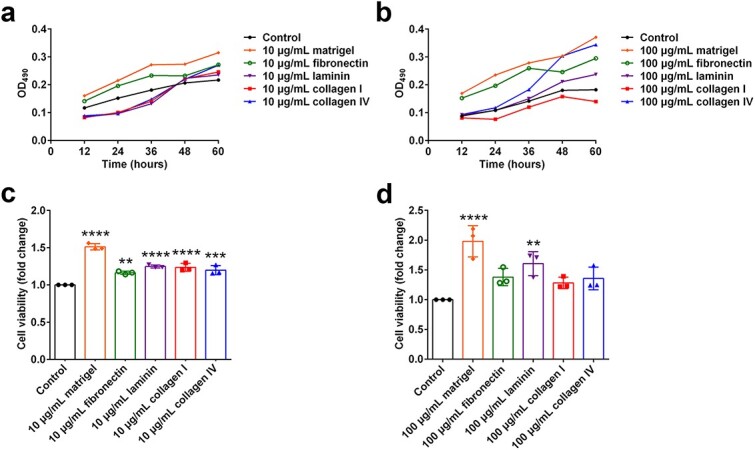
Effects of ECM components on Schwann cell growth. (**a**, **b**) Average growth curves of Schwann cells. *n* = 3 biological replicates. (**c**) Relative viability of Schwann cells grown on plates coated with 10 μg/ml Matrigel, fibronectin, laminin, collagen I, or collagen IV was normalized to that of the noncoated control. *n* = 3 biological replicates. *p* < 0.0001 for 10 μg/ml Matrigel, *p* = 0.0012 for 10 μg/ml fibronectin, *p* < 0.0001 for 10 μg/ml laminin, *p*< 0.0001 for 10 μg/ml collagen I, and *p* = 0.0002 for 10 μg/ml collagen IV *vs* the noncoated control. (**d**) Relative viability of Schwann cells grown on plates coated with 100 μg/ml Matrigel, fibronectin, laminin, collagen I, or collagen IV was normalized to that of the noncoated control. *n* = 3 biological replicates. *p* < 0.0001 for 100 μg/ml Matrigel, and *p* = 0.0041 for 100 μg/ml laminin *vs* the noncoated control. ^**^p < 0.01; ^***^p < 0.001; ^****^p < 0.0001. *ECM* extracellular matrix

To evaluate the effects of ECM components on Schwann cell growth, an MTT assay was performed, and the absorbance of the purple formazan solution was quantified as the mitochondrial dehydrogenases of viable cells transform MTT to formazan. The absorbance of formazan is directly related to the number of viable cells. The number of viable Schwann cells was observed 12 h after culture and the number of viable cells increased with time. The growth curves of Schwann cells cultured on Matrigel, fibronectin, laminin, collagen I, and collagen IV were generated and compared with the growth curves of Schwann cells cultured on noncoated control plates. Compared with those grown on noncoated plates or plates coated with ECM components, the growth rates of Schwann cells grown on 10 μg/ml Matrigel were greater. In addition to Matrigel, Schwann cells grown on 10 μg/ml fibronectin also exhibited better growth performance than those grown on noncoated plates (Figure 1a). Schwann cells also grew well on culture plates coated with a relatively high concentration of ECM components, i.e. 100 μg/ml. Like Schwann cells grown on 10 μg/ml ECM components, Matrigel and fibronectin strongly stimulated the growth of Schwann cells (Figure 1b).

The growth conditions of the Schwann cells were further examined via a CCK-8 assay. Consistent with the results of the MTT assay, Matrigel strongly increased the viability of Schwann cells. The application of 10 μg/ml Matrigel increased cell viability to >1.5-fold that of the noncoated control, whereas the application of 100 μg/ml Matrigel increased cell viability to nearly 2-fold that of the noncoated control. Other ECM components also increased cell viability (Figure 1c and d).

**Figure 2:**
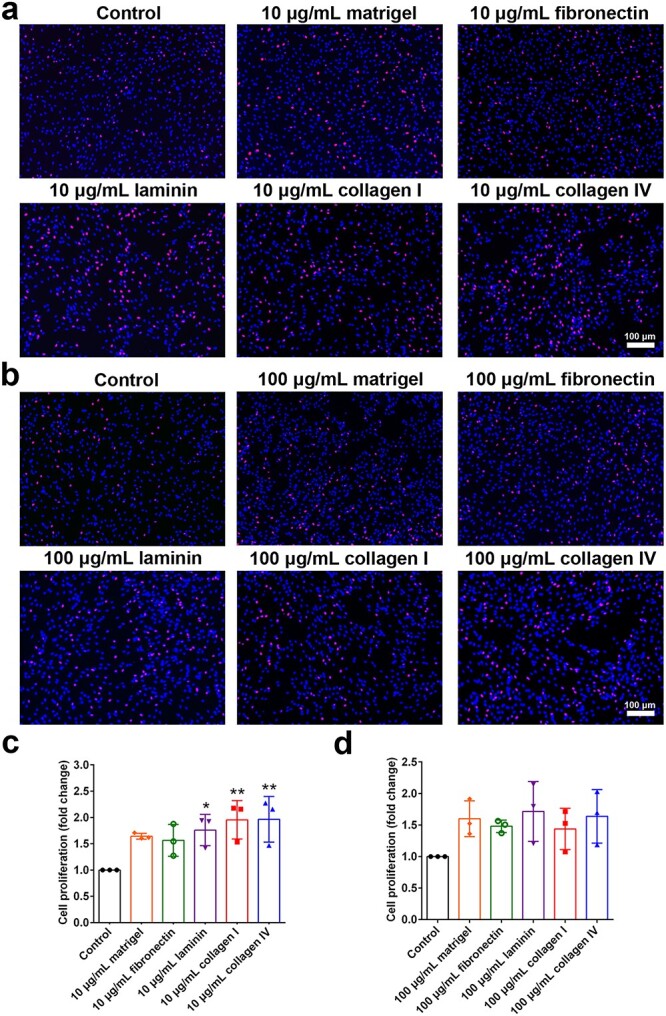
Effects of ECM components on Schwann cell proliferation. (**a**, **c**) Relative proliferation rates of Schwann cells grown on plates coated with 10 μg/ml Matrigel, fibronectin, laminin, collagen I, or collagen IV normalized to the noncoated control. Red represents EdU-positive cells and blue represents nucleus. *n* = 3 biological replicates. Scale bar: 100 μm. *p* = 0.0288 for 10 μg/ml laminin, *p* = 0.0068 for 10 μg/ml collagen I, and *p* = 0.0062 for 10 μg/ml collagen IV *vs* the noncoated control. (**b**, **d**) Relative proliferation rates of Schwann cells grown on plates coated with 100 μg/ml Matrigel, fibronectin, laminin, collagen I, or collagen IV normalized to the noncoated control. *n* = 3 biological replicates. Scale bar: 100 μm. ^*^p < 0.05; ^**^p < 0.01. *ECM* extracellular matrix

The proliferation of Schwann cells was assessed by incorporating EdU into newly synthesised DNA and measuring the number of DNA-synthesising cells. Compared with Schwann cells cultured on noncoated plates, a considerably greater number of EdU-positive Schwann cells cultured on plates coated with both 10 and 100 μg/ml ECM components were observed, suggesting the favourable role of ECM components, especially laminin, collagen I, and collagen IV, in Schwann cell proliferation (Figure 2).

### ECM components stimulate Schwann cell movement

**Figure 3:**
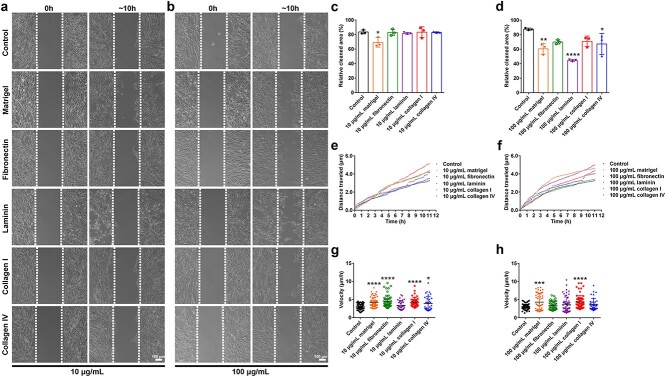
Effects of ECM components on Schwann cell movement. (**a**, **c**) Remaining cleaned areas of Schwann cells. *n* = 3 biological replicates. Scale bar: 100 μm. *p* = 0.0102 for 10 μg/ml Matrigel *vs* the noncoated control. (**b**, **d**) Remaining cleaned areas of Schwann cells. *n* = 3 biological replicates. Scale bar: 100 μm. *p* = 0.0033 for 100 μg/ml Matrigel, *p* < 0.0001 for 100 μg/ml laminin, and *p* = 0.0226 for 100 μg/ml collagen IV *vs* the noncoated control. (**e**) Average travel distances of Schwann cells. *n* = 3 biological replicates. (**f**) Average travel distances of Schwann cells. *n* = 3 biological replicates. (**g**) Movement velocities of Schwann cells. *n* > 35 Schwann cells. *p* < 0.0001 for 10 μg/ml Matrigel, *p* < 0.0001 for 10 μg/ml fibronectin, *p* < 0.0001 for 10 μg/ml collagen I, and *p* = 0.0134 for 10 μg/ml collagen IV *vs* the noncoated control. (**h**) Movement velocities of Schwann cells. *n* > 35 Schwann cells. *p* = 0.0002 for 100 μg/ml Matrigel and *p* < 0.0001 for 100 μg/ml collagen I *vs* the noncoated control. ^*^p < 0.05; ^**^p < 0.01; ^***^p < 0.001; ^****^p < 0.0001. *ECM* extracellular matrix

The influence of ECM components on the movement of Schwann cells was also investigated. Wound healing assay revealed that a small number of Schwann cells migrated towards the scratch ~10 h after removal of wound healing culture inserts. In Matrigel-coated plates as compared with noncoated plates or plates coated with other ECM components, more cells moved to the wound and thus the remaining cleaned area was relatively much smaller (Figure 3a and c). The movement of Schwann cells was stimulated when cultured on plates coated with higher concentrations of ECM components. Approximately 40% of the gap was filled with migrated Schwann cells in plates coated with 100 μg/ml Matrigel, leaving 60% of the wound area as blank space. The relative cleaned area also seemed to be smaller for Schwann cells cultured on plates coated with 100 μg/ml ECM components as compared with Schwann cells cultured on noncoated plates. Notably, for Schwann cells cultured on plates coated with 100 μg/ml laminin, the relative cleaned area was even smaller than that of cells cultured on Matrigel-coated plates, indicating the robust promoting effect of laminin on Schwann cell migration (Figure 3b and d).

The movement trajectory of Schwann cells was directly observed via live-cell imaging assay. The movement distances of Schwann cells cultured in cell chambers were calculated and displayed in line charts with each dot representing moved distances of Schwann cells at 10-min intervals during an 11 h observation. Average travelled distances showed that at a concentration of 10 μg/ml, in addition to Matrigel, fibronectin and collagen I motivated Schwann cell migration (Figure 3e). The summarised mean velocities of the Schwann cells also indicated the roles of fibronectin, collagen I, and collagen IV in promoting Schwann cell movement (Figure 3g). Schwann cells cultured on chambers coated with 100 μg/ml Matrigel and collagen I also seemed to have larger travelling distances (Figure 3f) and higher velocities (Figure 3h) as compared with cells cultured in noncoated chambers.

### ECM components promote neurite growth

**Figure 4:**
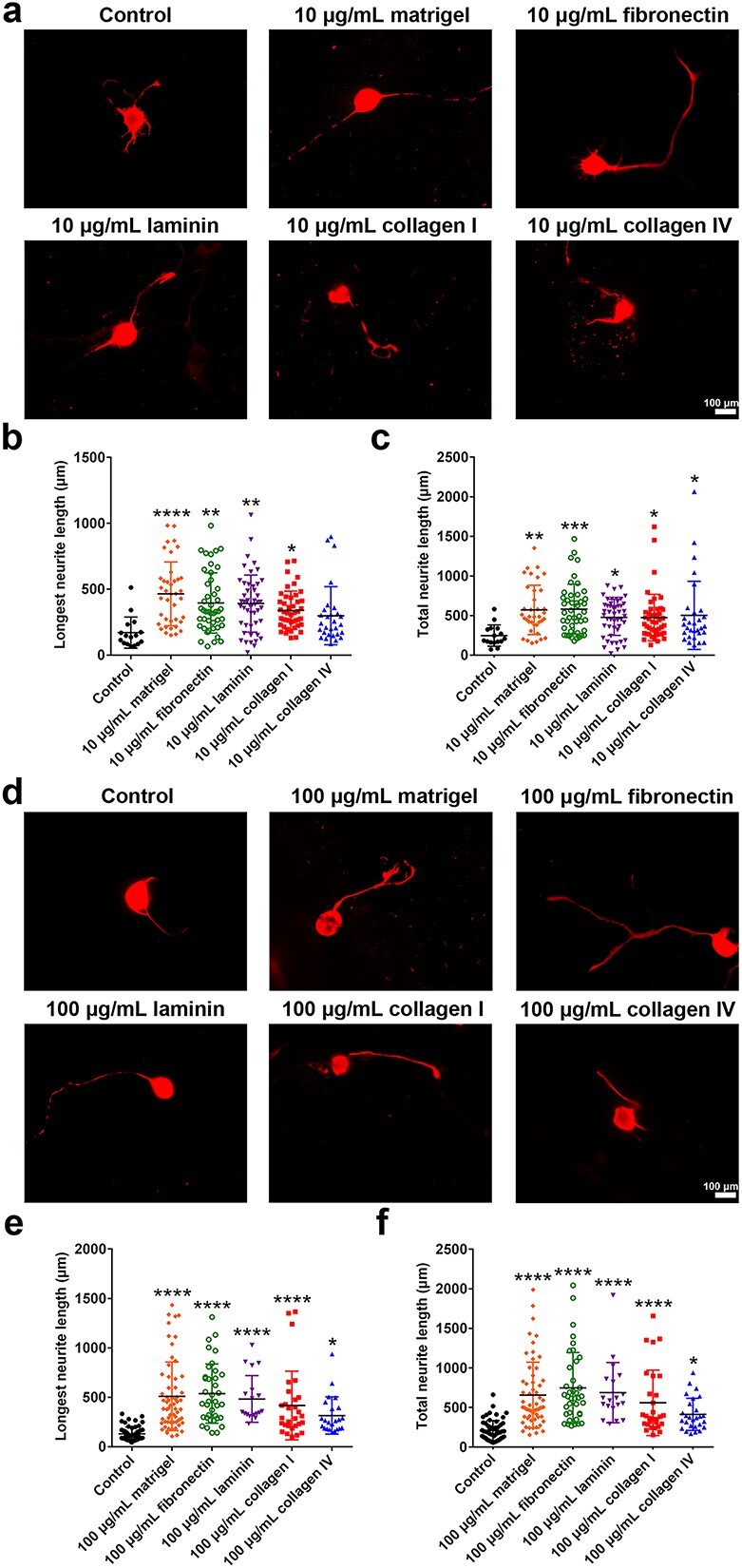
Effects of ECM components on neuron growth. (**a**) NF200 immunostaining of neurons. Scale bar: 100 μm. (**b**) Longest neurite length of neurons. *n* > 15 neurons. *p* < 0.0001 for 10 μg/ml Matrigel, *p* = 0.0010 for 10 μg/ml fibronectin, *p* = 0.0011 for 10 μg/ml laminin, and *p* < 0.0185 for 10 μg/ml collagen I *vs* the noncoated control. (**c**) Total neurite length of neurons. *n* > 15 neurons. *p* = 0.0016 for 10 μg/ml Matrigel, *p* = 0.0008 for 10 μg/ml fibronectin, *p* = 0.0300 for 10 μg/ml laminin, *p* = 0.0344 for 10 μg/ml collagen I, and *p* = 0.0238 for 10 μg/ml collagen IV *vs* the noncoated control. (**d**) NF200 immunostaining of neurons. Scale bar: 100 μm. (**e**) Longest neurite length of neurons. *n* > 15 neurons. *p* < 0.0001 for 100 μg/ml Matrigel, *p* < 0.0001 for 100 μg/ml fibronectin, *p* < 0.0001 for 100 μg/ml laminin, *p* < 0.0001 for 100 μg/ml collagen I, and *p* = 0.0291 for 100 μg/ml collagen IV *vs* the noncoated control. (**f**) Total neurite length of neurons. *n* > 15 neurons. *p* < 0.0001 *vs* the non coated control. ^*^p < 0.05; ^**^p < 0.01; ^***^p < 0.001; ^****^p < 0.0001. *ECM* extracellular matrix

Neurons were cultured on plates coated with various types of ECM components to determine the effects of ECM components on neuron behaviour. Representative immunostaining images revealed the robust role of ECM components in promoting neurite growth (Figure 4a). In summary, 10 μg/ml Matrigel increased the length of total neurites from ~245 μm in neurons in the noncoated control group to ~572 μm, and increased the length of the longest neurite from ~170 μm in neurons in the noncoated control group to ~465 μm. Treatment with 10 μg/ml fibronectin, laminin, collagen I, or collagen IV also led to increased total neurite length and the longest neurite length (Figure 4b and c). Neurons grown on plates coated with 100 μg/ml ECM components also presented longer neurite lengths (Figure 4d). Moreover, neurons cultured on plates coated with 100 μg/ml matrigel, fibronectin, laminin, or collagen I, but not collagen IV, had slightly longer neurites than neurons cultured on plates coated with the same proteins at the 10 μg/ml concentration (Figure 4e and f).

### Collagen I supports nerve regeneration by enhancing Schwann cell migration and vessel formation

**Figure 5:**
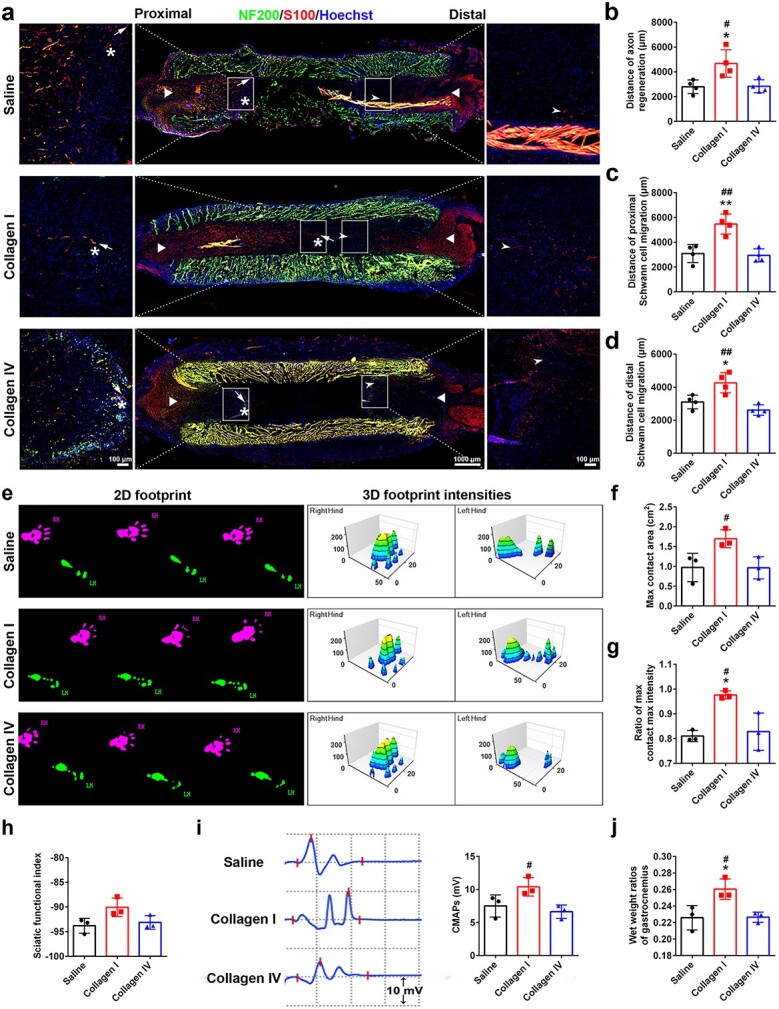
Effects of collagen I and collagen IV on Schwann cell migration and nerve regeneration. (**a**) NF200 and S100 immunostaining of longitudinal sections of regenerated sciatic nerves 14 days after gap bridging. Green indicates NF200 staining, red indicates S100 staining, and blue indicates Hoechst staining. Asterisks indicate the front edge of the regenerated axons. Arrows point to Schwann cells migrating from the proximal nerve stump, whereas arrowheads point to Schwann cells migrating from the distal nerve stump. Boxed areas are displayed at a higher magnification. Scale bars represents 1000 μm in the main image and 100 μm in the enlarged image. (**b**) Distance of regenerated axons located from the starting point of the proximal nerve stump to the front of extension 14 days after gap bridging. *n* = 4 rats. *p* = 0.0193 for collagen I *vs* saline and *p* = 0.0214 for collagen I *vs* collagen IV. (**c**) Distance of Schwann cell migration from the proximal nerve stump. *n* = 4 rats. *p* = 0.0024 for collagen l *vs* saline and *p* = 0.0016 for collagen I *vs* collagen IV. (**d**) Distance of Schwann cell migration from the distal nerve stump. *n* = 4 rats. *p* = 0.0155 for collagen I *vs* saline and *p* = 0.0018 for collagen I *vs* collagen IV. (**e**) Representative images of 2D and 3D footprint strengths 4 weeks after gap bridging. RH, right hind limb (normal side); LH, left hind limb (injury side). (**f**) Maximum contact area of the left hind limb. *n* = 3 rats. *p* = 0.0493 for collagen I *vs* collagen IV. (**g**) Ratio of max contact max intensity (LH/RH). *n* = 3 rats. *p* = 0.0110 for collagen I *vs* saline and *p* = 0.0184 for collagen I *vs* collagen IV. (**h**) Sciatic functional index values of each group. *n* = 3 rats. (**i**) Representative electromyography image of the injured side and compound muscle action potential (CMAP) amplitudes for each group at 4 weeks after gap bridging. *n* = 3 rats. *p* = 0.0367 for collagen I *vs* collagen IV. (**j**) Statistical results of the wet weight ratio of the gastrocnemius muscle 4 weeks after gap bridging. *n* = 3 rats. *p* = 0.0260 for collagen l *vs* saline and *p* = 0.0280 for collagen I *vs* collagen IV. ^*^p < 0.05 *vs* saline; ^**^p < 0.01 *vs* saline; #*p* < 0.05 *vs* collagen IV; ##*p* < 0.01 *vs* collagen IV

Considering the obvious role of collagen I in promoting Schwann cell activity and axon growth, collagen I was injected into chitosan and PLGA scaffolds to generate ECM component collagen I-based tissue-engineered nerve grafts. Collagen IV, another type of collagen, can also be injected into chitosan and PLGA scaffolds to bridge long-distance peripheral nerve injury. At 14 days after bridging, immunostaining for NF200 and S100 was performed on sciatic nerve sections to visualize axon and Schwann cell distributions, respectively (Figure 5a). Measurement of the length of regenerated axons revealed that in rats whose nerve gaps were bridged with saline-filled or collagen IV-filled scaffolds, injured axons grew for ~0.28 cm, whereas in the collagen I group, injured axons grew for a significantly shorter distance (Figure 5b). Compared with those in both the saline and collagen IV groups, the Schwann cells in the collagen I group also migrated longer distances from both the proximal and distal nerve stumps towards the injured site (Figure 5c and d). Postoperative gait assessments were conducted at 4 weeks after surgery. The footprints showed a notably greater maximum contact area for the injured hind paw in the collagen I group than in the collagen IV group (Figure 5e and f). The force applied by the injured hind paw to the glass surface in the collagen I group more closely resembled that of the normal side, outperforming the saline and collagen IV groups (Figures 5e and g), indicating a return of motor function. Although no significant biological differences were observed between the groups, the SFI value for the collagen I group was marginally greater than those for the saline and collagen IV groups (Figure 5h). The CMAPs indicate the quantity of nerve fibres innervating the target muscles. The peak amplitude of CMAPs in the collagen IV group did not differ biologically from that of the saline group, whereas the peak amplitude of CMAPs in the collagen I group was significantly greater (Figure 5i). Additionally, the wet weight ratio of the collagen I group markedly surpassed those of both the saline and the collagen IV groups (Figure 5j).

**Figure 6:**
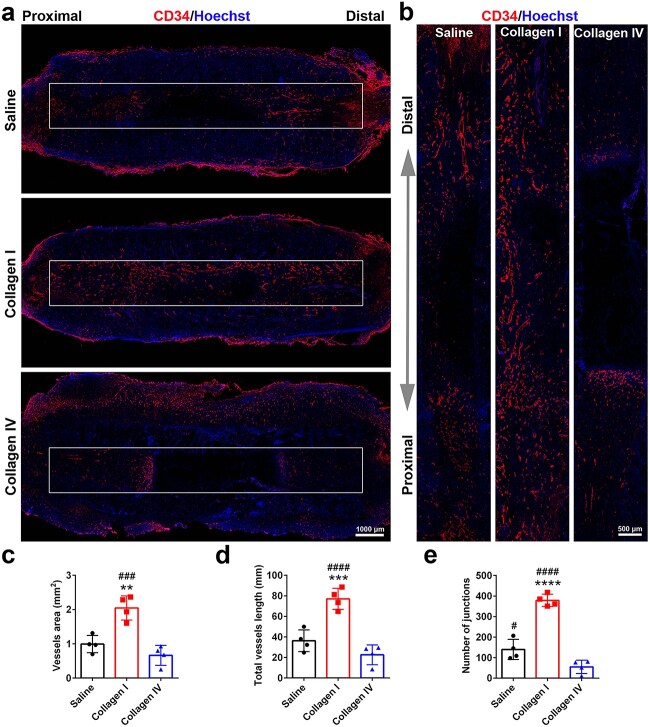
Effects of collagen I and collagen IV on blood vessel formation. (**a**, **b**) Immunostaining of the vessels inside the scaffold 14 days after gap bridging. Red indicates CD34 staining and blue indicates Hoechst staining. Boxed areas in (a) are displayed at a higher magnification in (b). Scale bars represents 1000 μm in (a) and 500 μm in (b). (**c**) Area of vessels inside the scaffold. *n* = 4 rats. *p* = 0.0020 for collagen I *vs* saline and *p* = 0.0003 for collagen I *vs* collagen IV. (**d**) Total length of vessels inside the scaffold. *n* = 4 rats. *p* = 0.0008 for collagen I *vs* saline and *p* < 0.0001 for collagen I *vs* collagen IV. (**e**) Number of junctions of vessels inside the scaffold. *n* = 4 rats. *p* < 0.0001 for collagen I *vs* saline, *p* = 0.0286 for collagen IV *vs* saline, and *p* < 0.0001 for collagen I *vs* collagen IV. ^**^p < 0.01 *vs* saline; ^***^p < 0.001 *vs* saline; ^****^p < 0.0001 *vs* saline; #*p* < 0.05 *vs* collagen IV; ###*p* < 0.001 *vs* collagen IV; ####*p* < 0.0001 *vs* collagen IV

In the collagen I group, increased blood vessel ingrowth from both proximal and distal nerve stumps was observed inside the scaffolds (Figure 6a and b), which was similar to the enhanced cell migration observed in cultured Schwann cells. Compared with saline- or collagen IV-injected rats, collagen I-injected rats had obviously larger areas and longer vessel lengths (Figure 6c and d). Correspondingly, an increased number of cellular junctions were observed in the collagen I group (Figure 6e), demonstrating that collagen I facilitates tubulogenesis and promotes neovascularization in the wound area.

### Collagen IV-induced fibroblast accumulation and excessive inflammation cause unfavourable outcomes

**Figure 7:**
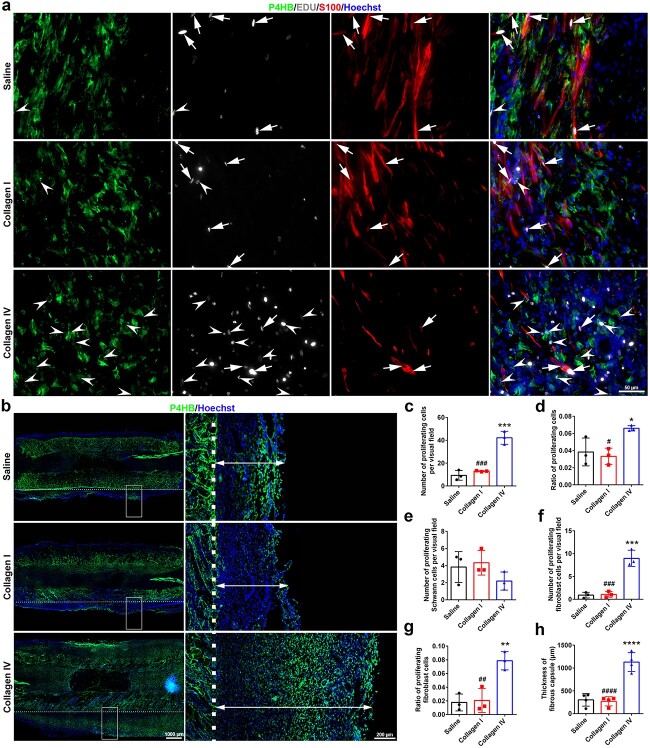
Effects of collagen I and collagen IV on fibroblast accumulation. (**a**) Immunostaining of fibroblasts inside the scaffold 14 days after gap bridging. Green indicates P4HB staining, white colour indicates EdU staining, red indicates S100 staining, and blue indicates Hoechst staining. Arrowheads point to EdU-positive fibroblasts. Arrows point to EdU-positive Schwann cells. Scale bar: 50 μm. (**b**) Immunostaining of fibroblasts outside the scaffold 14 days after gap bridging. Green indicates P4HB staining and blue indicates Hoechst staining. Boxed areas are displayed at a higher magnification. The dashed line indicates the outer edge of the scaffold. Scale bars represent 1000 μm in the main image and 200 μm in the enlarged image. (**c**) Number of EdU-positive proliferating cells. *n* = 3 rats. *p* = 0.0002 for collagen IV *vs* saline and *p* = 0.0003 for collagen I *vs* collagen IV. (**d**) Ratio of proliferating cells to total cells. *n* = 3 rats. *p* = 0.0495 for collagen IV *vs* saline and *p* = 0.0252 for collagen I *vs* collagen IV. (**e**) Number of S100- and EdU-positive proliferating Schwann cells. *n* = 3 rats. (**f**) Number of P4HB- and EdU-positive proliferating fibroblasts. *n* = 3 rats. *p* = 0.0003 for collagen IV *vs* saline and *p* = 0.0004 for collagen I *vs* collagen IV. (**g**) Ratio of proliferating fibroblasts to total fibroblasts cells. *n* = 3 rats. *p* = 0.0053 for collagen IV *vs* saline and *p* = 0.0067 for collagen I *vs* collagen IV. (**h**) Thickness of the fibrous capsule outside the scaffold. Three points at the proximal, middle, and distal segments on the same side of the longitudinal section of each nerve conduit were measured. *n* = 4 rats. *p* < 0.0001 for collagen IV *vs* saline and *p* < 0.0001 for collagen I *vs* collagen IV. ^*^p < 0.05 *vs* saline; ^**^p < 0.01 *vs* saline; ^***^p < 0.001 *vs* saline; ^****^p < 0.0001 *vs* saline; #*p* < 0.05 *vs* collagen IV; ##*p* < 0.01 *vs* collagen IV; ###*p* < 0.001 *vs* collagen IV; ####*p* < 0.0001 *vs* collagen IV

In addition to Schwann cells and endothelial cells, the wound microenvironment is composed of fibroblasts and various types of immune cells, especially macrophages. Coimmunostaining of sciatic nerve sections with the fibroblast marker P4HB, the Schwann cell marker S100, the proliferating marker EdU, and the nuclear marker Hoechst revealed strong P4HB- and EdU-positive signals in rats bridged with collagen IV-filled scaffolds (Figure 7a). Fewer proliferating cells were observed in the saline group and the collagen I group, whereas nearly 4.5-fold more proliferating cells were observed in the collagen IV group than in the saline group (Figure 7c). The ratio of proliferating cells to total cells was also greater in the collagen IV group although the number of total cells was also greater in the sciatic nerves of the rats after the transplantation of the collagen IV-filled scaffolds (Figure 7d). Quantification of cells costained with S100 and EdU revealed that although not significantly different, there was a slightly greater number of Schwann cells in the collagen I group. Moreover, although many cells were proliferating in the collagen IV group, the number of proliferating Schwann cells was not high (Figure 7e). On the other hand, the quantification of cells costained with P4HB and EdU revealed that many proliferating cells in the collagen IV group were fibroblasts (Figure 7f and g). The distribution of fibroblasts inside and outside the implanted scaffolds was then visualized in longitudinal sciatic nerve sections (Figure 7b). Redundant fibroblasts accumulated along the scaffold in the collagen IV group, forming a noticeably thicker fibrous capsule (Figure 7h).

**Figure 8:**
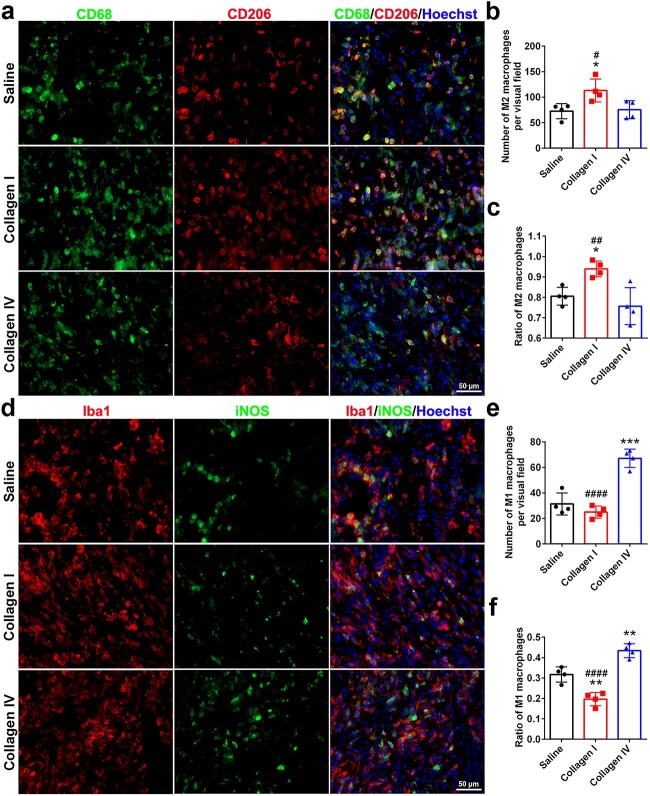
Effects of collagen I and collagen IV on macrophage invasion. (**a**) Immunostaining of M2 macrophages inside the scaffold 14 days after gap bridging. Green indicates CD68 staining, red indicates CD206 staining, and blue indicates Hoechst staining. The M2 macrophages were CD68-positive and CD206-positive. Scale bar: 50 μm. (**b**) Number of M2 macrophages. *n* = 4 rats. *p* = 0.0316 for collagen l *vs* saline and *p* = 0.0438 for collagen l *vs* collagen IV. (**c**) Ratio of M2 macrophages to total macrophages. *n* = 4 rats. *p* = 0.0344 for collagen l *vs* saline and *p* = 0.0064 for collagen I *vs* collagen IV. (**d**) Immunostaining of M1 macrophages inside the scaffold 14 days after gap bridging. Green indicates iNOS staining, red indicates Iba1 staining, and blue indicates Hoechst staining. The M1 macrophages were Iba1- and iNOS-positive. Scale bar: 50 μm. (**e**) Number of M1 macrophages. *n* = 4 rats. *p* = 0.0001 for collagen IV *vs* saline and *p* < 0.0001 for collagen l *vs* collagen IV. (**f**) Ratio of M1 macrophages to total macrophages. *n* = 4 rats. *p* = 0.0021 for collagen l *vs* saline, *p* = 0.0027 for collagen IV *vs* saline and *p* < 0.0001 for collagen I *vs* collagen IV. ^*^p < 0.05 *vs* saline; ^**^p < 0.01 *vs* saline; ^***^p < 0.001 *vs* saline; #*p* < 0.05 *vs* collagen IV; ##*p* < 0.01 *vs* collagen IV; ####*p* < 0.0001 *vs* collagen IV

Immunostaining of sciatic nerves with CD68 and Iba1, which is a pan marker of macrophages, revealed the invasion of macrophages after peripheral nerve injury (Figure 8a and d). Compared with those in the saline group, a greater number of macrophages seemed to be recruited to the injured sites after collagen injection. In the collagen I group, a majority of the recruited macrophages were CD206-positive M2 macrophages (Figure 8a–c). In the collagen IV group, more iNOS-positive M1 macrophages were recruited (Figure 8d–f). Notably, among the three groups, although there were more total macrophages in the collagen I group, the number and ratio of M1 macrophages seemed to be the lowest (Figure 8d–f).

**Figure 9:**
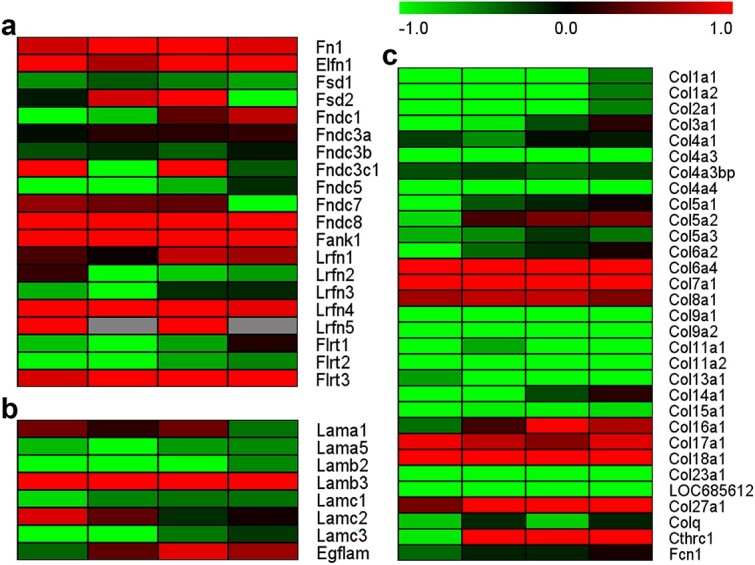
Temporal expression of genes encoding ECM components. (**a**) Relative expression of fibronectin-associated genes in rat sciatic nerves at 0, 1, 4, 7, and 14 days after nerve injury. Fn1, fibronectin 1; Elfn1, extracellular leucine-rich repeat and fibronectin type III domain containing 1; Fndc8, fibronectin type III domain containing 8; Fank1, fibronectin type 3 and ankyrin repeat domains 1; Lrfn4, leucine-rich repeat and fibronectin type III domain containing 4; and Flrt3, fibronectin leucine-rich transmembrane protein 3 were consistently upregulated. (**b**) Relative expression of laminin-associated genes in rat sciatic nerves at 0, 1, 4, 7, and 14 days after nerve injury. Lamb3, laminin, and beta 3 were consistently upregulated. (**c**) Relative expression of collagen-associated genes in rat sciatic nerves at 0, 1, 4, 7, and 14 days after nerve injury. Col6a4, collagen, type VI, alpha 4; Col7a1, collagen, type VII, alpha 1; Col8a1, collagen, type VIII, alpha 1; Col17a1, collagen, type XVII, alpha 1; Col18a1, collagen, type XVIII, alpha 1; and Col27a1, collagen, type XXVIII, alpha 1 were consistently upregulated. Red represents upregulation, green represents downregulation, and grey represents undetected. *ECM* extracellular matrix

## Discussion

ECM components significantly regulate the phenotypes of various types of cells. For example, collagen I, an ECM component that is positively related to renal cell carcinoma development, reportedly increases the migration of renal cell carcinoma cells and enhances tumour cell invasion [25]. Collagen coating does not influence the motility of fibroblasts but changes their shape to a rounder structure [26]. The effects of the ECM on Schwann cells have also been explored. Laminin signalling promotes Schwann cell survival and proliferation, whereas collagen signalling promotes Schwann cell adhesion, spreading, and myelination [27,28].

In a recent study, polydimethylsiloxane was incubated with 10 μg/ml collagen I, fibronectin, or laminin solution, the cellular properties were quantified, and the results revealed that collagen I, fibronectin, and laminin induced increased 5-bromodeoxyuridine (BrdU) incorporation [29]. In our recent study, in addition to collagen I, fibronectin, and laminin, we also coated plates/chambers with collagen IV and compared the effects of these ECM proteins. A relatively high concentration of ECM proteins, i.e. 100 μg/ml, was also applied. Consistent with the observations of others [29], we found that these ECM components increased EdU incorporation, suggesting the beneficial effects of ECM components on Schwann cell proliferation. In addition to measuring DNA synthesis with BrdU or EdU incorporation assays, the proliferation of Schwann cells was further investigated via the cellular metabolism method, with CCK-8 and MTT as markers. The cellular metabolism method determines the number of living cells, whereas the DNA synthesis method traces cell cycle kinetics and quantifies cells in the G1, S, and G2/M phases [30,31]. Our study revealed the enhancing effects of ECM proteins on cellular metabolism, indicating that the increase in cell viability is concentration dependent. These results provide a more comprehensive view of the effects of ECM components on the proliferation of Schwann cells.

In addition to the proliferation of Schwann cells, the migration of Schwann cells is also essential for the formation of nerve bridges between nerve stumps and subsequent axon pathfinding during peripheral nerve regeneration [32,33]. Here, via a wound-healing assay and live-cell imaging, we investigated the beneficial effects of ECM components on Schwann cell movement. Accelerated Schwann cell migration may facilitate the reconnection of nerve stumps and thus contribute to peripheral nerve regeneration. Measurement of the length of neurites from neurons cultured on ECM component-coated plates clearly demonstrated the role of the ECM component in promoting neuron growth.

Nevertheless, these different ECM components display diverse cellular responses. Collagen I, as the major structural protein, promotes Schwann cell proliferation and migration as well as neurite outgrowth and thus was combined with chitosan and PLGA scaffolds to construct tissue-engineered nerve guidance conduits [34] to explore the *in vivo* effects of collagen I on peripheral nerve regeneration. Revascularization is a fundamental element for nerve repair and a key factor that should be considered during the construction of tissue-engineered nerve grafts [9,35]. Newly formed blood vessels function as tracks to guide the migration of Schwann cells, whereas migrated Schwann cells direct the oriented growth of injured axons [36,37]. Our immunostaining results revealed that after collagen I implantation, polarized blood vessels concatenate to bridge the nerve gaps and the tissue-engineered nerve grafts are fully vascularized. Following the polarized vasculature, in the collagen I group, Schwann cells migrated a greater distance towards the wound area and guided the elongation of axons.

Unlike collagen I, the implantation of collagen IV, another type of collagen that promotes Schwann cell activity and axon growth *in vitro*, did not improve Schwann migration and Schwann cell-mediated axon regeneration. This finding suggested that in addition to the permissive regeneration microenvironment generated by endothelial cells and Schwann cells, some adverse factors may exist at the injured sites. Notably, abundant fibroblasts were observed to proliferate and accumulate inside and outside of the tissue-engineered nerve grafts after the addition of collagen IV. Collagen IV, the main component of the basement membrane, strongly interacts with fibroblasts *in vivo*. Fibroblasts mediate extracellular matrix remodelling and are critical for tissue repair and wound healing [38,39]. However, excessive fibroblasts, especially inflammatory fibroblasts, may induce scar formation and pose hurdles to successful regeneration [40,41]. Compared with fibroblast heterogeneity, the functional heterogeneity of macrophages has been recognised for an even longer time period, and M1/M2 macrophages can be more easily distinguished by specific markers [42]. Immunostaining with macrophage-specific markers revealed the infiltration of more proinflammatory M1 macrophages but fewer anti-inflammatory M2 macrophages after the addition of collagen IV. The switching of macrophages from the M1 phenotype to the M2 phenotype is beneficial for peripheral nerve regeneration [43]. Therefore, collagen I-induced M2 macrophages may be helpful for nerve repair, whereas collagen IV-induced M1 macrophages may be harmful. The *in vivo* results indicated that even without considering the abundance of each component in the extracellular matrix, their roles are unique. Collagen I is the protagonist that supports and guides nerve cell regeneration and is more suitable for the construction of artificial nerve grafts. Collagen IV is also important, but may be more critical in the maturation and remodelling stages of nerve regeneration. These observations provide a more comprehensive view of the functions of diverse cell types in the wound microenvironment and provide a basis for the use of environmental cues to orchestrate the generation of tissue-engineered nerve grafts.

The abundance and composition of the ECM are modified under various physiological and pathological conditions. Our previous high-throughput analyses revealed dynamic changes in the levels of multiple matrix metalloproteinases, key enzymes for ECM component cleavage and ECM remodelling, after sciatic nerve injury [44]. The gene encoding matrix metalloproteinase 9 has even been recognised as one of the most significantly dysregulated genes in injured sciatic nerves [45,46]. The robust changes in matrix metalloproteinases imply that many ECM components may be differentially expressed. Therefore, the changes in the expression of genes encoding ECM components after peripheral nerve injury were explored on the basis of previous sequencing analysis [23]. Many fibronectin-associated genes, including fibronectin 1 (Fn1), extracellular leucine-rich repeat and fibronectin type III domain containing 1 (Elfn1), fibronectin type III domain containing 8 (Fndc8), fibronectin type 3 and ankyrin repeat domains 1 (Fank1), leucine rich repeat and fibronectin type III domain containing 1/4/5 (Lrfn1/4/5), and fibronectin leucine rich transmembrane protein 3 (Flrt3), were upregulated in the injured peripheral nerves (Figure 9a). However, for laminin-associated genes, only laminin, gamma 3 (Lamc3) was elevated at all test time points after nerve injury, whereas laminin, alpha 5 (Lama5), laminin, beta 2 (Lamb2), and laminin, gamma 3 (Lamc3) were downregulated (Figure 9b). Similarly, the expression levels of most collagen-associated genes decreased following peripheral nerve injury, with only collagen, type VI, alpha 4 (Col6a4), collagen, type VII, alpha 1 (Col7a1), collagen, type VIII, alpha 1 (Col8a1), Col17a1 (collagen, type XVII, alpha 1), collagen, type XVIII, alpha 1 (Col18a1), and collagen, type XXVIII, alpha 1 (Col27a1) remaining elevated in injured nerves (Figure 9c). Because of the low abundance of collagen I in the injured nerve stumps and the positive role of collagen I in microenvironment reconstruction and nerve regeneration, combining collagen I with tissue-engineered nerve grafts and applying exogenous collagen I to the injured site may be helpful for the treatment of peripheral nerve injury.

## Conclusions

Taken together, the results of our study illuminate the modulatory effects of ECM components on cell populations in peripheral nerves, reveal essential regulatory factors involved in peripheral nerve regeneration, and identify collagen I as a promising biomaterial for the construction of tissue-engineered nerve grafts.

## Abbreviations

ECM: Extracellular matrix; MTT: methylthiazolyldiphenyl-tetrazolium bromide; Edu: 5-ethynyl-2'-deoxyuridine; PLGA: Poly (lactic-co-glycolic acid); SD: Sprague–Dawley; DMEM; dulbecco's modified eagle medium; FBS; fetal bovine serum; BSA: bovine serum albumin; DMSO: dimethyl sulfoxide; IgG: immunoglobin G; Cy3: cyanine 3; iNOS: inducible nitric oxide synthase; NCBI: national center for biotechnology information; RPKM: Reads per kilobase per million mapped reads; ANOVA: Analysis of variance; SFI: Sciatic functional index; CMAPs: Compound muscle action potentials; Brdu: 5-bromodeoxyuridine.

## Data Availability

The datasets used and/or analyzed in the current study are available from the corresponding author upon reasonable request.

## Founding

This work was supported by grants from the National Key R&D Program of China (2022YFC2409800、2022YFC2409802), the Postgraduate Research & Practice Innovation Program of Jiangsu Province (KYCX23_3381), the Natural Science Foundation of Jiangsu Province (BK20231338), Jiangsu Provincial Key Medical Center and Priority Academic Program Development of Jiangsu Higher Education Institutions (PAPD), Jiangsu College Students Innovation and Entrepreneurship Training Program (202310304120Y), and the Science and Technology Plan Program of Nantong (MS2023050).
